# Transcriptional Responses of *Lacticaseibacillus rhamnosus* to TNFα, IL-6, IL-8, and IL-10 Cytokines

**DOI:** 10.3390/biology13110931

**Published:** 2024-11-15

**Authors:** Ksenia M. Klimina, Marina S. Dyachkova, Vladimir A. Veselovsky, Natalia V. Zakharevich, Aleksandra A. Strokach, Oksana V. Selezneva, Egor A. Shitikov, Dmitry A. Bespiatykh, Roman A. Yunes, Elena U. Poluektova, Maya V. Odorskaya, Polina S. Ostroukhova, Sergey A. Bruskin, Valeriy N. Danilenko, Evgenii I. Olekhnovich

**Affiliations:** 1 Lopukhin Federal Research and Clinical Center of Physical-Chemical Medicine of Federal Medical Biological Agency, Malaya Pirogovskaya Str. 1a, Moscow 119435, Russia; 2 Vavilov Institute of General Genetics, Russian Academy of Sciences, Moscow 119991, Russia

**Keywords:** commensal gut microbiota, immunomodulatory bacteria, host immune system response, cytokine signaling, RNA sequencing, transcriptome, phylogenetic profiling, phosphotransferase system

## Abstract

The interaction between gut microbiota and the host immune system is a complex and understudied field, with cytokines like TNFα, IL-6, IL-8, and IL-10 playing pivotal roles. Commensal bacteria, including lactobacilli, respond to these cytokines through adaptive mechanisms that support their survival and function within the gut. While the influence of cytokines on pathogenic bacteria is well documented, their impact on commensal bacteria, particularly lactobacilli, remains underexplored. This study investigates the transcriptional responses of *Lacticaseibacillus rhamnosus* strains K32 and R19-3 to various cytokines using next-generation RNA sequencing (RNA-seq). Our findings reveal that cytokines, especially IL-8 and IL-10, significantly alter the *L. rhamnosus* transcriptome, affecting genes involved in carbohydrate metabolism, stress response, and transcriptional regulation. Notably, IL-8 and IL-10 induce a significant downregulation of genes related to the phosphotransferase system, suggesting a reduction in metabolic activity in response to inflammatory signals. This study unveils a previously unexplored aspect of *L. rhamnosus* adaptation, highlighting its intricate response to cytokine signals. By modulating gene expression, *L. rhamnosus* may mitigate the adverse effects of inflammation and promote gut health. These insights could inform the development of targeted probiotic therapies for inflammatory bowel disease (IBD) and other conditions with altered cytokine levels. Our results suggest that co-evolution between a host and gut microbiota enables bacteria to respond to specific cytokines through gene expression changes, revealing a unique and underexplored facet of the interaction between commensal bacteria and the host organism.

## Simple Summary

The gut microbiota plays a pivotal role in sustaining human health, especially through its interactions with the immune system. This study examines how the bacterium *Lacticaseibacillus rhamnosus* responds to cytokines, which are essential for controlling inflammation and are well studied in relation to pathogenic bacteria. However, less is understood about the response of commensal bacteria like *L. rhamnosus* to these immune factors. Using RNA sequencing, we analyzed the genetic responses of four *L. rhamnosus* strains to various cytokines, including TNFα, IL-6, IL-8, and IL-10. Our findings indicate that cytokines significantly alter bacterial gene expression, particularly in pathways linked to energy metabolism and stress response. This research advances our understanding of how these bacteria adapt to immune cues, supporting gut health. The results could lead to novel probiotic therapies, offering potential treatments for inflammatory diseases.

## 1. Introduction

The human gastrointestinal tract constitutes a complex and diverse ecosystem that harbors a myriad of microorganisms collectively known as the gut microbiota (GM). GM plays a pivotal role in influencing various physiological processes, encompassing nutrient metabolism and immune system modulation and ultimately contributing to human health and well-being [1]. Interactions between the bacteria of the GM and the host’s immune are reciprocal, wherein the GM facilitates antigenic stimulation, modulates phagocytosis, influences the production of immunoglobulin A and cytokines, and impacts the differentiation of immunocompetent cells [2]. This intricate interplay between the GM and the immune system is of considerable scientific significance due to its profound implications for host health [3,4]. One mechanism underpinning this interaction involves responses to the signaling molecules of the immune system, such as peptide hormones and neurotransmitters, as evidenced by numerous studies [5]. Notably, certain bacteria, particularly opportunistic human pathogens, possess the capability to bind host cytokines, leading to heightened growth, formation of biofilms, and alterations in virulence characteristics [6,7,8,9,10,11,12]. Such processes, when occurring within the human body, often culminate in inflammation.

Unlike pathogenic bacteria, commensal GM bacteria interact with the host organism’s immune system without eliciting inflammation, a phenomenon known as homeostatic immunity [13]. The mechanisms enabling commensal microorganisms to uphold and safeguard the equilibrium of the normal GM while navigating inflammatory conditions remain poorly understood. Our study aimed to address this gap by exploring the transcriptional responses of *Lacticaseibacillus rhamnosus*, a widely studied probiotic, to key immune cytokines. Previously, we demonstrated that the commensal strain of the human intestinal microbiota *B. longum* GT 15 alters gene transcription patterns in response to the cytokine TNFα [14].

*L. rhamnosus* (formerly known as *Lactobacillus rhamnosus*), although not autochthonous in the classical sense, exhibits adaptations to various ecosystems, including invertebrate and vertebrate hosts as well as food materials, rendering it ‘nomadic’ [14]. This species is generally recognized as safe (GRAS) by the U.S. Food and Drug Administration (FDA). Certain strains within this species demonstrate beneficial effects on the human body, such as antioxidant, anti-inflammatory, and immunogenic properties [15,16]. They are capable of surviving in the gastrointestinal tract, possess strong adhesion capabilities, and may be classified as probiotics. *L. rhamnosus* stands out as one of the most prevalent and extensively studied probiotic species, representing approximately 10% of the species involved in health claims applications [17]. These factors make *L. rhamnosus* an ideal model organism for investigating the mechanisms underlying beneficial host–microbe interactions.

Next-generation RNA sequencing technology is increasingly utilized to investigate the bacteria of the *Lactobacillaceae* family, opening up new avenues in genetic variability analysis. For instance, studies have examined global transcriptomic changes in *Lactobacillus acetotolerans* F28 [18] and *Lacticaseibacillus casei* SMN-LBK [19] under ethanol exposure. Additionally, the transcriptome of *Lactobacillus bulgaricus* 2038 in various growth phases [20], the influence of lactoferrin on the growth of *Lactiplantibacillus plantarum* GG at low temperature [21], and the resistance of *L. plantarum* ZDY2013 to acid and osmotic stress [22] were examined. The impact of acid stress on *L. rhamnosus* GG was also studied [23]. These studies underscore the significance of transcriptomic analysis as a potent tool for elucidating cellular changes under the influence of diverse factors.

In this study, we, for the first time, explored the adaptation mechanisms of *L. rhamnosus* strains to the presence of cytokines TNFα, IL-6, IL-8, and IL-10. The transcriptional profiles of *L. rhamnosus* K32 under the influence of these cytokines were investigated, revealing distinctive features of cytokine impact. Furthermore, by employing phylogenetic profiling and transcriptomic analysis, we made significant progress in predicting the genetic pathways of *L. rhamnosus* K32 that contribute to its resistance against immune response factors during inflammatory processes.

## 2. Materials and Methods

### 2.1. Bacterial Strain and Growth Condition

In this work, the *Lactobacillus* strains *L. rhamnosus* K32 (GenBank JNNV00000000), *L. rhamnosus* R19-3 (LWBT00000000), 40f (LFNA00000000), and 116 (JTDC00000000) were used. Specifically, *L. rhamnosus* K32 (GenBank JNNV00000000) was isolated in 2010 from the gastrointestinal tract of a healthy adult female in Central Russia. *L. rhamnosus* R19-3 (LWBT00000000) was isolated in 2011 from a fecal sample of a healthy 13-year-old girl from the Aktobe region of Kazakhstan. The strain 40f (LFNA00000000) was isolated in 2010 from a fecal sample of a 19-year-old healthy woman in Central Russia, and strain 116 (JTDC00000000) was originally isolated in 2014 from the feces of a healthy adult female in Central Russia. Cultivation was carried out in Lactobacillus MRS Broth (HiMedia, Mumbai, Maharashtra, India) at 37 °C under anaerobic conditions (HiAnaerobic System—Mark III, AnaeroHiGas Pack 3.5L; HiMedia, Mumbai, Maharashtra, India).

### 2.2. The Effect of Pro-Inflammatory and Anti-Inflammatory Cytokines on the L. rhamnosus Strain

The strain’s overnight culture was diluted at a ratio of 1:100 in 2 mL of medium. Experimental samples underwent treatment with lyophilized recombinant human cytokines, namely, IL-6, TNFα, IL-8, and IL-10, at concentrations of 0.1, 1, and 10 ng/mL, respectively, procured from Thermo Fisher Scientific (Waltham, MA, USA). To ensure accuracy, a control group was established, comprising the same *L. rhamnosus* strain grown in MRS medium supplemented with deionized water and recombinant green fluorescence protein (GFP) (# A42613) from Thermo Fisher Scientific (USA). This control facilitated the assessment of any impurities present in the recombinant cytokines. Optical density (OD600) measurements were performed using the SmartSpec Plus spectrophotometer (BIO RAD, Hercules, CA, USA), and growth curves were constructed over a 24 h period. Transcriptomic analysis was meticulously conducted during the bacterial culture’s exponential growth phase (OD600 = 1.2). The experiment was rigorously performed in triplicate to ensure the reliability and robustness of the results.

### 2.3. RNA Extraction

Total RNA was extracted from a 2 mL cell culture, initially treated with 1 mL of RNAprotect Bacteria Reagent (Qiagen, Hilden, Germany) to stabilize the RNA. Cell disruption occurred in 2 mL Lysing Matrix B tubes using a MagNA Lyzer device (Roche, Mannheim, Germany) for 30 s. For RNA extraction, we utilized an automatic station, KingFisher Flex (Thermo Fisher Scientific, USA), in conjunction with the MagMAX™ mirVana™ Total RNA Isolation Kit (Thermo Fisher Scientific, USA), following the manufacturer’s protocols. The quantity and quality of isolated RNA were assessed using a Qubit device with the Quant-it RiboGreen RNA assay kit (Thermo Fisher Scientific, USA) and a Bioanalyzer 2100 equipped with the RNA 6000 Pico chip (Agilent Technologies, Santa Clara, CA, USA), respectively.

Total RNA underwent additional treatment with the TURBO DNA-free kit (Thermo Fisher Scientific, USA) in a 50 μL volume. Subsequent purification of RNA was carried out using Agencourt RNA Clean XP kit magnetic particles (Beckman Coulter, Brea, CA, USA). Quantity and quality assessments of the isolated total RNA were reiterated using a Qubit device with the Quant-it RiboGreen RNA assay kit (Thermo Fisher Scientific, USA) and a Bioanalyzer 2100 equipped with the RNA 6000 Pico chip (Agilent Technologies, USA), respectively.

### 2.4. Preparation of Transcriptomic Libraries and RNA Sequencing

For the preparation of transcriptomic libraries, 200 ng of total RNA was taken as input. Selective removal of ribosomal RNA was performed using the Ribo-Zero Plus rRNA Depletion Kit (Illumina, San Diego, CA, USA), followed by library preparation employing the NEBNext^®^ Ultra II Directional RNA Library Prep Kit (NEB), in accordance with the manufacturer’s instructions. Subsequent RNA purification steps involved the application of RNA Clean XP magnetic beads (Beckman Coulter, Brea, CA, USA), with final library purification conducted using Agencourt AMPure XP magnetic beads (Beckman Coulter, Brea, CA, USA). The size distribution and quality of the libraries were assessed using the Agilent High Sensitivity DNA kit (Agilent Technologies, USA), while the library concentration was quantified using the Quant-iT DNA Assay Kit, High Sensitivity (Thermo Fisher Scientific, USA). Following this, the libraries were equimolarly pooled and diluted to a final concentration of 12 pM. Sequencing of the prepared libraries was executed on an Illumina HiSeq 2500 platform utilizing the HiSeq Rapid SBS Kit v2 (50 cycles) and the HiSeq SR Rapid Cluster Kit v2 for single reads, supplemented with 1% Phix (Illumina) as an internal control.

### 2.5. Data Processing and Analysis

Quality control of the reads was conducted using FastQC v0.12.1 [24]. Subsequently, the reads were aligned to the reference genome employing HISAT2 v2.2.1 [25]. Quality assessment of the mapped reads was performed using Qualimap v.2.2.2-dev [26]. Conversion of files from SAM to BAM format, along with their sorting and indexing, was achieved using SAMtools v1.18 [27]. Read counting aligned with genomic annotations was conducted using featureCounts v2.0.6 [28]. Differential gene expression analysis was carried out utilizing the edgeR 3.40.2 package for R v4.1.2 [29]. Genes exhibiting an FDR value of less than 0.05 and a change in expression level exceeding 2-fold were deemed differentially expressed. Statistical analysis for growth curve construction was performed using Microsoft Office Excel 2019. Heatmap generation utilized the R-packages ggplot2 v3.4.4 [30] and tidyHeatmap v0.1.0 [31]. DEGs were functionally characterized by the Cluster of Orthologous Genes (COG) [32] employing the reCOGnizer v1.10.1 tool [33].

### 2.6. Phylogenetic Profiling

Phylogenetic profiling comprised several steps: First, ortholog groups were identified for *Lacticaseibacillus* genomes. Next, binary vectors were constructed to indicate presence (1) or absence (0) across multiple genomes. Subsequently, a matrix of pairwise distances between these vectors was created, followed by the grouping of phylogenetic profiles (PP). This process utilized OrthoFinder v.2.5.4 [34]. PPs were generated for 163 complete genomes of *Lacticaseibacillus* representatives, which included *L. rhamnosus* K32, alongside genomic contigs of *L. rhamnosus* R19. Pairwise distances between PPs were calculated using the Jaccard Similarity Metric. Genes were grouped based on minimal PP pairwise distances (cutoff = 0.001) in the distance matrix, physical proximity (within 10,000 bp) within the same strand, and co-regulation of all genes within the groups. For this analysis, a custom Python v.3.10.2 script was employed (accessible at https://github.com/LabGenMO/phylo-profiling/blob/master/Identification%20of%20connected%20genes.ipynb, accessed on 16 November 2020).

### 2.7. Quantitative Real-Time PCR

Primers listed in Table 1 were used to assess the mRNA levels of the target genes. For qPCR, the 2FRT PCR mixture containing Mg^2+^ ions (Amplisens, Moscow, Russia) was used, along with the thermostable TaqF DNA polymerase (Amplisens, Moscow, Russia); a 25× nucleotide mix (Thermo Scientific, USA); a 20× intercalating dye, EVA Green (Biotium, Fremont, CA, USA); a reference dye, ROX (Sintol, Moscow, Russia); and synthesized primers for target and control genes (Evrogen, Moscow, Russia) (Table 1). The prepared reaction mixtures of 8 μL were added to a 96-well plate, followed by the addition of 2 μL (~10 ng) of the template (cDNA) to each well, and the plate was sealed tightly with film. Each sample was run in triplicate. The qPCR was performed on an Applied Biosystems 7500 Real-Time PCR System (Life Technologies, Carlsbad, CA, USA) using the RQ 1.2 software (Relative Quantitation software, Life Technologies, USA). The following thermal cycling protocol was used: initial denaturation and enzyme activation at 95 °C for 15 min; 50 cycles of denaturation for 15 s at 95 °C, primer and probe annealing; and elongation for 1 min at 60 °C. The data obtained were analyzed using control genes and the relative quantification or ΔΔCt method (Table 1). All calculations were performed using the Gene Transcription Analysis (GTA) application (Certificate No. 2008612585, 2008, Rospatent, RF), compatible with the software of Applied Biosystems 7000/7500 series amplifiers [35,36]. This program was developed for evaluating the relative mRNA levels in paired samples using multiple control genes and allows the determination of PCR efficiency. Taking into account the variability of control genes, expression changes were considered significant if they were 2-fold or greater.

## 3. Results

### 3.1. Investigating the Impact of Pro-Inflammatory and Anti-Inflammatory Cytokines on L. rhamnosus K32 Growth

**Figure 1:**
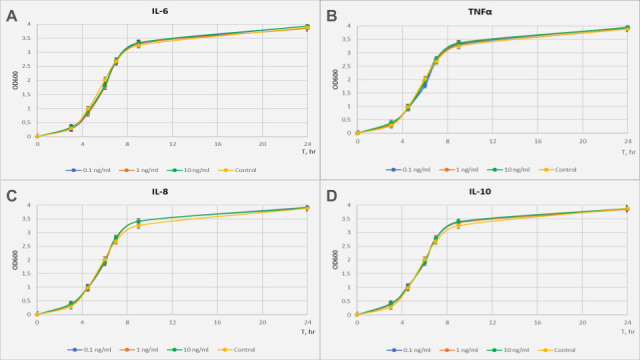
Growth curves of *L. rhamnosus* K32 strain with the addition of cytokines at concentrations of 0.1 ng/mL, 1 ng/mL, and 10 ng/mL to the medium: (**A**) IL-6, (**B**) TNFα, (**C**) IL-8, (**D**) IL-10.

We selected *L. rhamnosus* K32 as a model organism due to its well-documented probiotic properties and adaptability to diverse environments, including the human gastrointestinal tract [37]. To assess the effect of pro-inflammatory (IL-6, IL-8, TNFα) and anti-inflammatory (IL-10) cytokines on bacterial culture growth, *L. rhamnosus* K32 was incubated with various concentrations of cytokines, and OD600 values were measured at different time points. In our study, we used concentrations of the cytokines TNF-α, IL-6, IL-8, and IL-10 based on previous research, which demonstrated that concentrations of 1 ng/mL of these cytokines can affect the growth of pathogenic strains such as *Staphylococcus aureus*, *Pseudomonas aeruginosa*, and *Acinetobacter* spp. [8]. Although such concentrations are rarely found in the human body, they are used in certain therapeutic approaches for cancer treatment, confirming their biological significance and justifying our choice of doses for the experiments. Growth curves of *L. rhamnosus* K32 were constructed based on OD600 measurements in experimental and control conditions (Figure 1).

The experiment showed that the presence of IL-6, IL-8, IL-10, and TNFα in the growth medium did not affect the culture growth rate. However, these cytokines may influence the expression of genes unrelated to cell growth. Therefore, the same cultures were used for further transcriptomic analysis.

### 3.2. Advancing Understanding of Lactobacillus Response to Cytokines Through Transcriptome Sequencing

To deepen our understanding of the underlying mechanisms of *Lactobacillus*’ response to cytokines, we conducted high-throughput transcriptome sequencing of the *L. rhamnosus* K32 strain during the exponential growth phase under both experimental and control conditions using Illumina technology, across three independent biological replicates. The number of reads obtained from sequencing is presented in Supplementary Table S1.

For the primary analysis, we used Plot to assess the convergence of points between experiments (Supplementary Figure S1). Consequently, experimental data for IL-6 and IL-8 at a concentration of 0.1 ng/μL were excluded from the analysis. Genes exhibiting significant differential expression (FC ≥ |1.5|, FDR < 0.05) compared to the control (deionized water) in response to cytokine presence were identified (Supplementary Table S2). Transcriptomic analysis revealed no differentially expressed genes (DEGs) in response to IL-6 in *L. rhamnosus* K32. However, a significant number of DEGs were identified under the influence of other cytokines (TNFα, IL-8, and IL-10), particularly with IL-8 and IL-10 (Table 2, Supplementary Table S2).

It is worth noting that cytokine exposure at varying concentrations (0.1 ng/mL, 1 ng/mL, and 10 ng/mL) impacts the number of DEGs but not the expression level of these genes (Table 3).

Table 2 demonstrates that a consistent expression profile was observed across all cytokine concentrations, suggesting that the cytokines’ effect was not dose-dependent for the *L. rhamnosus* K32 strain. Based on this observation, a concentration of 1 ng/mL was selected for subsequent experiments. The expression levels of five genes (QJQ50_RS06580, QJQ50_RS06020, QJQ50_RS03685, QJQ50_RS04870, QJQ50_RS09120) were confirmed by quantitative PCR, indicating the reliability of the obtained data. However, recent studies demonstrated that RNA-seq data, in many cases, can provide reliable insights without the need for additional validation through qPCR [38].

### 3.3. Differences in Gene Transcription Profiles

In the investigation of the mechanisms underlying *Lactobacilli*’s response to inflammatory processes, an analysis of changes in transcription profiles under the influence of recombinant cytokines was conducted. Using RNA-seq technology from Illumina, the transcriptomes of the *L. rhamnosus* K32 strain at the exponential growth phase were analyzed. The experiment was performed in three biological replicates. Samples were cultured in two groups: experimental (with the addition of cytokines IL-8, IL-10, TNFα, and IL-6 at a concentration of 1 ng/mL) and control (with the addition of GFP at a concentration of 1 ng/mL). The choice of using GFP as a control was justified by its ability to minimize the impact of impurities in the recombinant proteins, as even 1% impurities can significantly influence the results of transcriptional analysis. This is particularly significant because lipopolysaccharide contamination in recombinant proteins isolated from *E. coli* can cause the production of various cytokines and chemokines, similar to the response to inflammation [39].

**Figure 2:**
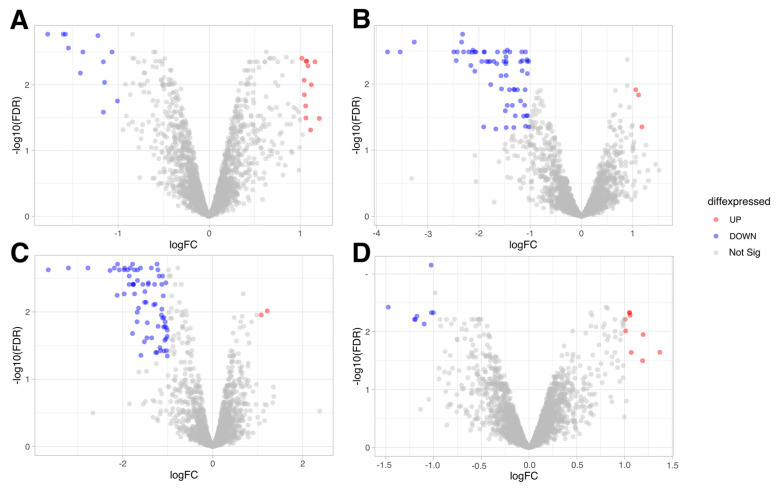
Global DEGs in *L. rhamnosus* K32 resulting from (**A**) exposure to IL-6, (**B**) exposure to IL-8, (**C**) exposure to IL-10, (**D**) exposure to TNFα compared to the GFP (FC ≥ |2|, FDR < 0.05). Red dots: upregulated genes (UP); blue dots: downregulated genes (DOWN); gray dots: not significant genes (Not Sig).

Following sequencing, a total of 23,571,644, 20,412,590, 15,241,304, and 23,615,467 unique reads were obtained for samples cultured with cytokines IL-8, IL-10, TNFα, and IL-6, and the GFP, respectively. After filtering out low-quality reads, the number of effective reads matching the genome of the *L. rhamnosus* K32 strain decreased to 23,507,790, 20,352,745, 15,199,420, and 23,550,106, respectively. A subsequent analysis identified genes whose differential expression significantly changed in response to the presence of cytokines (FC ≥ |2|, FDR < 0.05). DEGs were detected in *L. rhamnosus* K32 samples cultured with various cytokines compared to control samples. Their numbers are presented in Figure 2 and Supplementary Table S3.

From the data illustrated in Figure 2, it is evident that the highest number of genes was activated when the *L. rhamnosus* K32 strain was cultured in the presence of cytokines IL-8 (3 upregulated and 54 downregulated genes (Figure 2B)), and IL-10 (2 upregulated and 70 downregulated genes (Figure 2C)). At the same time, the addition of IL-6 (12 upregulated and 12 downregulated genes (Figure 2A)) and TNFα (9 upregulated and 8 downregulated genes (Figure 2D)) to the culture medium also led to a change in the expression profile of the *L. rhamnosus* K32 strain: however, these gene expression changes did not exceed a 2-fold increase. In contrast, the effects of cytokines IL-8 and IL-10 resulted in a change in gene expression levels by up to 14-fold (according to data from Table S3). Under the influence of IL-8 and IL-10, changes in the expression profile of strain *L. rhamnosus* K32 were associated with a decrease in the expression of 54 and 70 genes, respectively. In contrast, the impact of cytokines TNFα and IL-6 on the expression profile of *L. rhamnosus* K32 was significantly lower. Based on these observations, our work focuses on studying the effects of IL-8 and IL-10 on the *L. rhamnosus* K32 strain.

**Figure 3:**
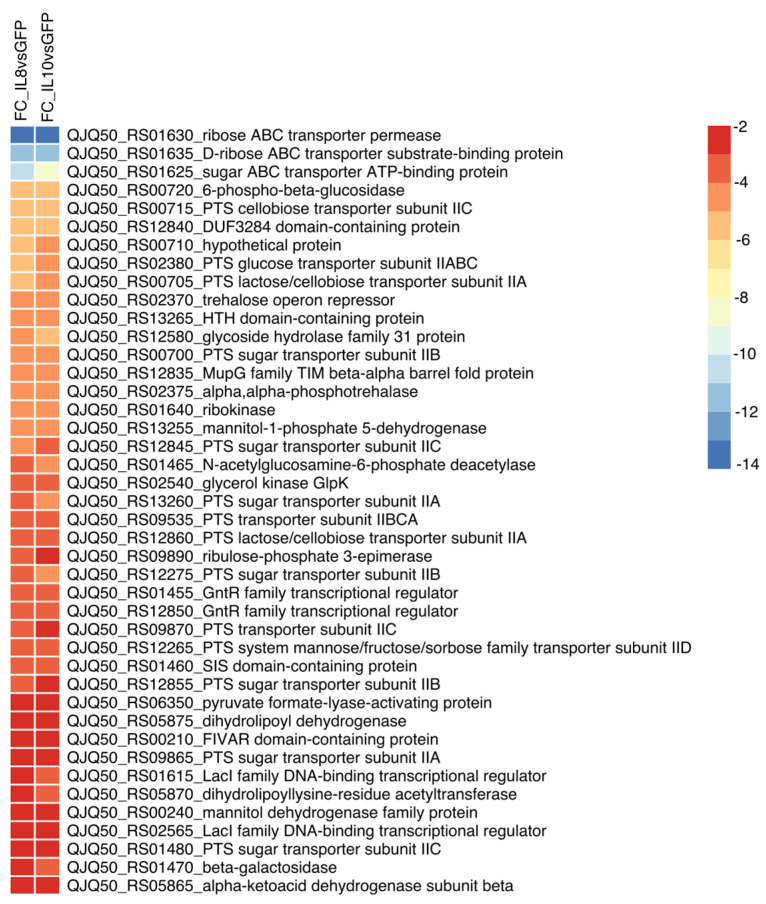
Heatmap of commonly differentially expressed genes (DEGs) between IL-8 and IL-10 treatments in *L. rhamnosus* K32 strain (FC ≥ |2|, FDR < 0.05).

Under the influence of cytokines IL-8 and IL-10, a decrease in the expression of the majority of genes (54 and 70, respectively) was observed. In particular, the transcription of genes for sugar metabolism was reduced. Specifically, the levels of expression of genes associated with the phosphotransferase system (PTS) of sugar transport, ABC transporters of sugars, and genes involved in galactose and mannitol metabolism were reduced. Additionally, there was a reduction in the expression of various transcriptional regulators, including those of the GntR family transcriptional regulators and LacI family DNA-binding transcriptional regulators (Figure 3). It is worth noting that common genes’ transcription reduction was identified for these two cytokines (Figure 3).

Figure 3 illustrates that DEGs associated with the PTS, responsible for sugar transport into cells, predominated among genes with reduced expression.

### 3.4. Functional Annotation of DEGs

**Figure 4:**
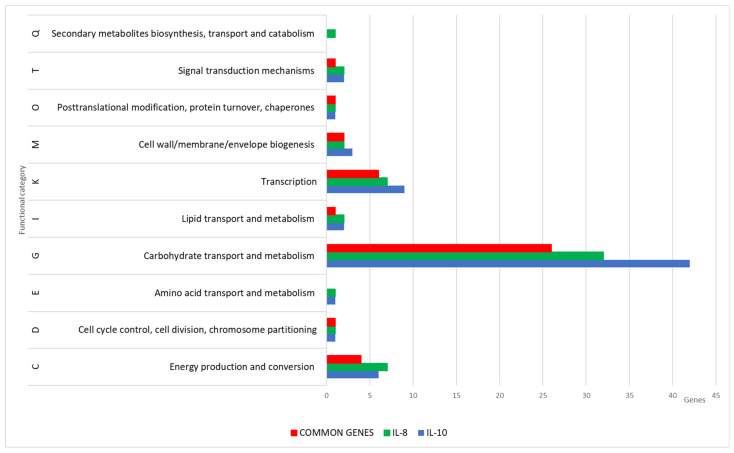
Functional classification of *L. rhamnosus* K32 DEGs with statistically significant decreases in mRNA levels compared to the GFP control.

The DEGs were then classified into functional groups based on the Clusters of Orthologous Groups (COG) classification system. The COG categories, including K (transcription), G (carbohydrate transport and metabolism), and C (energy production and conversion), exhibited a significant number of genes with reduced expression following the cultivation of the strain with IL-10 and IL-8, compared to the GFP control. Additionally, these groups showed a high number of DEGs common to both IL-8 and IL-10. Furthermore, for IL-10, a gene (QJQ50_RS00105) exhibited upregulation of its expression belonging to the category V (defense mechanisms), while for IL-8, a gene (QJQ50_RS04340) showed upregulation of its expression belonging to the category M (cell wall/membrane/envelope biogenesis). Figure 4 shows DEGs that exhibited downregulated expression in response to IL-10 and IL-8 treatment.

The changes in the expression profile, characterized by decreased expressions associated with carbohydrate transport and metabolism, energy production and conversion, and transcription, suggested a reduction in the metabolic activity of *L. rhamnosus* K32 in response to cytokines IL-8 and IL-10, indicating a shift to a protective state.

### 3.5. Evolutionarily Stable Gene Groups and Transcriptional Organization of DEGs

To identify putative operons within DEGs, we employed phylogenetic profiling—a method leveraging the co-occurrence or co-absence of traits across diverse *Lacticaseibacillus* species to infer functional associations (Supplementary Table S4). In essence, gene pairs exhibiting highly similar phylogenetic profiles, with a Jaccard distance threshold of less than 0.001, located within 10,000 nucleotides on the same genomic strand, and demonstrating concordant differential expression (either co-upregulated or co-downregulated) compared to the control, were categorized as putative operons. We analyzed the differential expression data relative to the control under two conditions: IL-8 treatment and IL-10 treatment.

**Figure 5:**
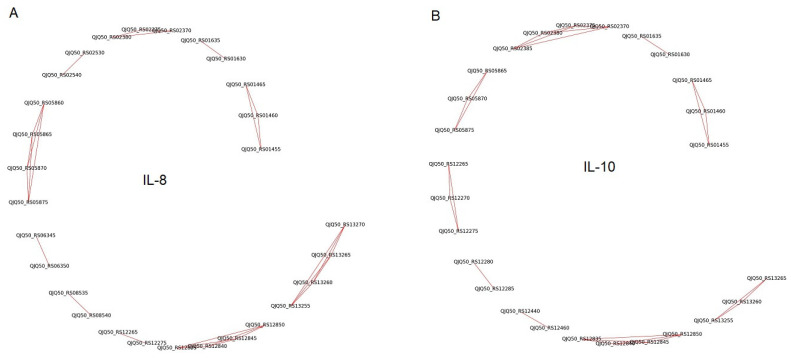
Operons of *L. rhamnosus* K32 identified using the phylogenetic profiling method, whose gene expression depends on the presence of cytokines (**A**) IL-8 and (**B**) IL-10 in the medium.

An analysis of the IL-8-treated samples through the lens of differential expression data revealed 10 putative operons containing two or more functionally linked genes within the *L. rhamnosus* K32 strain (Supplementary Table S5, Figure 5). Similarly, an analysis of the IL-10-treated samples identified 9 putative operons (Supplementary Table S5, Figure 5). The majority of these identified putative operons exhibited concordant downregulation in both IL-8 and IL-10 treatment experiments compared to the control. Notably, our analysis highlighted a significant proportion of functionally associated gene clusters in *L. rhamnosus* K32, which experienced downregulation following treatment with both IL-8 and IL-10 and were affiliated with the PTS. This system is well characterized for its involvement in carbohydrate substrate uptake and utilization. Furthermore, apart from the pyruvate metabolic pathway, a pivotal component of anaerobic glucose fermentation and energy metabolism, our analysis unveiled putative operons linked to ribose and trehalose utilization. Specifically, treatment with IL-8 alone resulted in the synchronous downregulation of genes within putative operons associated with glycerol catabolism. Additionally, a decline in the expression of operons in the pyruvate metabolic pathway, particularly those specific to the lactic acid bacteria, was observed. Conversely, IL-10 treatment alone led to the synchronous downregulation of genes within putative operons associated with carbohydrate catabolism and utilization.

### 3.6. The Impact of IL-8 and IL-10 on L. rhamnosus R19-3

Based on the obtained results, we tested the hypothesis whether the influence of cytokines IL-8 and IL-10 on gene expression in the *L. rhamnosus* K32 strain is unique to the strain or characteristic of other *L. rhamnosus* species. The other strains selected for evaluating the cytokines’ effects were the *L. rhamnosus* R19-3, 116, and 40f. The selection of strains was due to the availability of a sequenced genome, which enabled a comprehensive transcriptomic analysis (GenBank LWBT00000000.1, LFNA00000000, JTDC000000000). To examine the impact of IL-8 and IL-10 cytokines on bacterial culture growth, the strains were incubated with cytokine concentrations of 1 ng/mL, using GFP at a concentration of 1 ng/mL as a control. Among several tested *L. rhamnosus* strains, only the R-19 strain exhibited significant DEGs in response to cytokine exposure.

Sequencing yielded a total of 4,651,831, 4,173,819, and 4,484,246 unique reads for samples cultured with cytokines IL-8, IL-10, and the GFP, respectively. After filtering out low-quality reads, the number of effective reads mapped to the genome of the *L. rhamnosus* R19-3 strain decreased to 4,530,015, 4,078,397, and 4,384,335, respectively.

An analysis of the *L. rhamnosus* R19-3 sample cultured with IL-8 revealed 74 DEGs compared to the control sample, including 61 genes with decreased expression and 13 genes with increased expression (FC ≥ |2|, FDR < 0.05). For the *L. rhamnosus* R19-3 strain cultured with IL-10, 51 DEGs were detected compared to the control sample, among which 37 genes showed decreased expression and 14 genes exhibited increased expression (FC ≥ |2|, FDR < 0.05) (see Supplementary Table S6, Figure S2).

**Figure 6:**
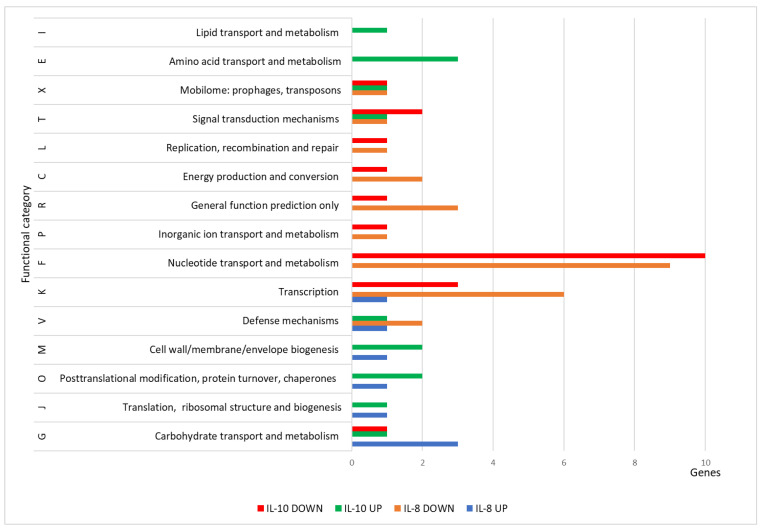
Functional classification of *L. rhamnosus* R19-3 DEGs with statistically significant decreases in mRNA levels compared to the GFP control.

The impact of cytokines on the *L. rhamnosus* R19-3 strain appeared to be less pronounced compared to the effects observed in the *L. rhamnosus* K32 strain. However, a notable similarity in the effects of IL-8 and IL-10 could be discerned, particularly characterized by a decrease in the expression of genes associated with the functional category of transcription (Figure 6). Additionally, there was a reduction in the expression of genes involved in nucleotide transport and metabolism. Conversely, an increase in the expression of genes related to cell wall/membrane/envelope biogenesis and posttranslational modification, protein turnover, and chaperones was observed.

In the case of strain R19, employing phylogenetic profiling akin to the methodology used for strain K32 enabled the identification of three clusters. These clusters exhibited co-downregulation and shared similar phylogenetic profiles (see Supplementary Table S5, Figure S3). The putative operons identified, likely involved in purine biosynthesis and amide uptake, exhibited reduced expression upon exposure to both IL-8 and IL-10. In contrast, a putative operon likely involved in the cell wall D-alanylation pathway exhibited increased expression upon exposure to both IL-8 and IL-10.

## 4. Discussion

In our previous research on the *B. longum* GT15 strain, we found that the introduction of cytokines IL-6 and TNFα into the culture medium had a minimal impact on its growth rate. However, these cytokines did affect the expression of specific genes [40]. Consequently, we chose to incorporate these cytokines into the experiments involving the *L. rhamnosus* strains. Numerous studies demonstrated the ability of *L. rhamnosus* to regulate the production of cytokines IL-8 and IL-10. Therefore, based on a thorough review of the literature, [41,42,43,44,45,46,47], we selected these cytokines to explore their impact on the *L. rhamnosus* K32 strain. Consequently, this study focused on four cytokines: IL-8, IL-10, TNFα, and IL-6 [48,49,50,51,52,53].

DEG analysis for the *L. rhamnosus* K32 strain revealed that the effects of cytokines IL-8, IL-10, and TNF-α at various concentrations (0.1, 1, and 10 ng/mL) were not dose-dependent. Despite alterations in cytokine concentration, similar changes in gene expression were observed. This phenomenon may be attributed to the ability of even minimal cytokine amounts to bind to receptors and elicit biological responses [54]. Such findings could explain why increasing cytokine concentration does not modify the expression level of the affected DEGs.

Certain bacteria, primarily opportunistic human pathogens, are able to bind host cytokines [55]. The known bacterial cytokine-binding proteins represent a rather versatile group of proteins, including a channel-forming usher protein [56], a Gram-negative secretin [12], an outer membrane pore protein [57], etc. Previously, a gene responsible for encoding a protein with motifs homologous to the cytokine-binding region of the human gp-130 receptor was identified in the genomes of commensal bacteria from the *Bifidobacterium* genus; the presence of genes encoding cytokine-binding-like structures in the genomes of commensals suggests that these microorganisms may also be able to perceive such signals [57]. The capability of the FNIII domains of the protein described above to selectively bind to the cytokine TNFα was experimentally validated in *B. longum*. We demonstrated that the presence of the pro-inflammatory cytokines IL-6 and TNFa in the growth medium of the *B. longum* subsp. *longum* GT15 strain affected the expression of certain genes [40].

The absence of genes homologous to the *fn3* gene in *L. rhamnosus* strains, compared to *B. longum*, explains the limited effect of TNFα on the *L. rhamnosus* K32 strain. The fact that we observed the effects of the cytokines IL-8 and IL-10 and minimal effects of TNFα on *L. rhamnosus* strain K32 suggests that the sensory system for human cytokines in lactobacilli is different from that of bifidobacteria.

Changes in the expression profile of the *L. rhamnosus* K32 strain in response to the introduction of cytokines IL-8 and IL-10, especially the reduction in the expression of genes associated with the phosphotransferase system, indicate that these cytokines influence the bacterium’s metabolic pathways related to carbohydrate assimilation. Additionally, the PTS serves as a complex protein kinase system that regulates a wide range of transport, metabolic, and mutagenic processes, as well as the expression of numerous genes. The decrease in the expression of genes involved in the phosphotransferase system, responsible for carbohydrate assimilation, may suggest that bacterial cells transition into a state of “minimal interaction with the environment”, possibly to conserve resources or as a protective response to inflammatory signals. It may also be part of the bacterium’s mechanism of interacting with the host immune system, wherein altering metabolism reduces detectability or modulates the immune response. The connection between PTS and bacterial resistance to antibiotics was demonstrated. Disabling or reducing PTS activity leads to increased resistance, which may serve as a protective mechanism during inflammation in response to cytokines [58].

Furthermore, changes in the expression of transcriptional regulators, such as regulators of the GntR and LacI families, may exert additional influence on the regulation of various metabolic pathways and the cell’s response to external conditions. These regulators play a role in controlling the expression of numerous genes, influencing bacterial adaptation to changes in the external environment.

Studying the presumed operons among the differentially expressed genes (DEGs) of the *L. rhamnosus* K32 strain allows for a deeper understanding of how groups of genes coordinately respond to the influence of cytokines IL-8 and IL-10, shedding light on their role in cellular processes and inflammation. The expression of an operon responsible for glycerol catabolism under aerobic conditions via glycerol kinase activity was downregulated. Additionally, the expression of operons involved in D-ribose transport and utilization, trehalose metabolism, pyruvate metabolism, and the phosphotransferase system for sugar transport into the cell was decreased. Overall, there was a reduction in the uptake of many sugars, suggesting a transition to minimal interaction with the external environment and a restructuring of metabolic pathways to obtain alternative sources of carbohydrates and energy. Although we may not observe a decrease in culture growth rate due to these changes, as the culture grows in a nutrient-rich environment, under conditions of competitive resource allocation within the organism, the growth rate is likely reduced.

The *L. rhamnosus* R19-3 strain exhibited a slightly different response to cytokines IL-8 and IL-10. The operons affected by these cytokines are related to the synthesis of teichoic acids, to the uptake of extracellular amide, which is hydrolyzed in the cytoplasm, and purine biosynthesis. Overall, this may indicate a slowdown in the growth rate and activation of protective mechanisms under conditions of inflammation.

The detection of these gene groups in a single operon under the influence of IL-8 and IL-10 suggests that, in response to these cytokines, bacteria may regulate key biochemical pathways. This could have significant implications for their metabolism, stress response, and interaction with the host immune system. In turn, this may contribute to bacterial adaptation to the inflammatory environment or modulation of the inflammatory process.

Based on the above, it can be argued that the response of the *L. rhamnosus* K32 strain was stronger than that of the *L. rhamnosus* R19-3 strain to the addition of cytokines IL-8 and IL-10 to the medium. However, in the case of *L. rhamnosus* R19-3, these cytokines affected the expression of PTS genes, indicating a shared influence of cytokines on *L. rhamnosus*. A similar response to the introduction of cytokines IL-8 and IL-10 can be explained by their collaborative action during inflammation. IL-8 acted as a chemokine and participated in attracting neutrophils, which contribute to the maintenance of commensal microbiota, compartmentalization, and separation of commensal bacteria during the invasion of pathogenic bacteria [59].

While extensive research has explored the impact of probiotics on host gene expression, comparatively little is known about how probiotic bacteria themselves respond to host-derived signals, such as inflammatory cytokines. Our findings align with a limited number of studies that demonstrated the transcriptional responses of other probiotic and commensal bacteria to inflammatory signals.

Specifically, studies with *B. longum* GT15 revealed gene expression changes in response to TNFα, akin to our observations with *L. rhamnosus* K32 [40]. Thus, similar to *L. rhamnosus*, *B. longum* exhibited a complex transcriptional response to cytokines, suggesting a common adaptive strategy among commensal bacteria. However, specific gene expression patterns may vary between species, reflecting their unique ecological niches and metabolic capabilities. Such responses highlight a broader pattern among probiotics, where adaptation to stress conditions might be mediated through modulation of metabolic and stress response pathways, as seen in *L. rhamnosus strain GG* [24]. These adaptations enable commensal and probiotic bacteria, such as *L. rhamnosus*, to survive and maintain their beneficial functions, including immune modulation, in stressful conditions like inflammation. This suggests that *L. rhamnosus* strains, with their ability to adapt to inflammatory signals, could be promising candidates for targeted probiotic therapies in immune-mediated inflammatory diseases. For example, strains that exhibit specific transcriptional responses to cytokines could be prioritized for use in conditions with altered cytokine levels. Specifically, conditions such as inflammatory bowel disease (IBD) and celiac disease are characterized by an increased expression of the cytokine genes IL-8 and IL-17A in intestinal biopsy specimens [60]. IBD also features elevated IL-6 gene expression, and TNF expression is increased in intestinal biopsies from patients with irritable bowel syndrome [61]. It is likely that elevated levels of these cytokines can also be detected in the intestinal lumen, as cytokines are secreted by epithelial cells both basolaterally and apically, as demonstrated by in vitro [62,63] and in vivo studies [64].

These insights are consistent with evidence from multi-strain probiotics, such as VSL#3, which have shown anti-inflammatory effects through modulation of the TLR4-NF-κB signaling pathway in murine models [65,66]. Clinical studies on VSL#3 demonstrated efficacy in maintaining remission in ulcerative colitis, suggesting that similar formulations could be further investigated in human models to evaluate their effectiveness in inflammatory diseases [67,68,69].

In conclusion, the results of this study underscore the complexity of the interaction between bacterial cells and their environment, highlighting the role of cytokines in modulating bacterial metabolism. This finding is significant for understanding microbial adaptation and its potential impact on the host.

## 5. Conclusions

Our findings provide valuable insights into the transcriptional responses of *L. rhamnosus* strains K32 and R19-3 to cytokines, revealing the complex interplay between gut microbiota and the host immune system. The observed transcriptional changes suggest that these commensal bacteria employ adaptive mechanisms to maintain homeostasis and resist inflammatory conditions. Furthermore, understanding the molecular mechanisms underlying the interaction between probiotic strains and the host immune system could aid in designing engineered probiotics with enhanced survival and functional potential.

## Figures and Tables

**Table 1:** Oligonucleotides used in this study.

Genes	Forward Primer	Reverse Primer	Product Length
QJQ50_RS06580	TCGACTGAAGATAAAGGGCACA	CGCTACTTTTCGTGCCGATG	131
QJQ50_RS06020	TTATCGCTGGGAATGCTTGC	CGTGCATGGCACTTAAGATCA	79
QJQ50_RS09120	GATTACGCATCTGCTGCACG	ACTGCCTTGATCACCCGTTT	80
QJQ50_RS03685	GGCCAGTTAGCCATCAGCAA	ATTGGTTTTTCGGCTGGCAC	80
QJQ50_RS04870	ACTGGCAAGGTAGTAGGCGT	CGTCCTCCTGTTTGATTGGC	70

**Table 2:** DEGs in *L. rhamnosus* K32 influenced by cytokines (FC ≥ |1.5|, FDR < 0.05).

Cytokines	Concentration of Cytokines, ng/mL	Number of DEGs
IL-6	0.1	-
	1	0
	10	0
IL-8	0.1	-
	1	190
	10	89
IL-10	0.1	75
	1	98
	10	123
TNFα	0.1	34
	1	6
	10	14

**Table 3:** Impact of cytokine concentration on the expression profile of shared genes of *L. rhamnosus* K32 under exposure to IL-10 and IL-8 cytokines (FDR < 0.05).

*L. rhamnosus* K32	10 ng/mL		1 ng/mL		0.1 ng/mL
GeneID	IL-10	IL-8	IL-10	IL-8	IL-10
QJQ50_RS03835	−1.614876	−1.667862	−1.653061	−1.731752	−1.539934
QJQ50_RS04815	1.688911	1.584739	1.510946	1.539842	1.551358
QJQ50_RS05005	1.611521	1.614288	1.531467	1.588289	1.511980
QJQ50_RS06985	1.606293	1.836675	1.684830	1.774548	1.579407
QJQ50_RS08010	−1.523734	−1.595751	−1.599999	−1.569289	−1.526031
QJQ50_RS11360	1.888934	1.723103	1.823643	1.761015	1.760617

## Data Availability

The raw Illumina transcriptome sequencing data of *L. rhamnosus* K32, *L. rhamnosus* R19-3, *L. rhamnosus* 40f, and *L. rhamnosus* 116 were deposited in NCBI under the accession numbers PRJNA1082667, PRJNA1082662, PRJNA1132985, and PRJNA1132986, respectively.

## Supplementary Materials

The following supporting information can be downloaded at https://www.mdpi.com/article/10.3390/biology13110931/s1: Supplementary Table S1. Total reads from RNA sequencing of *L. rhamnosus* K32 treated with different cytokines. Supplementary Table S2. Lists of DEGs in *L. rhamnosus* K32 strain treated with cytokines (IL-6, TNFα, IL-8, IL-10) at 0.1, 1, 10 ng/mL concentrations in the medium compared to the control (deionized water). Supplementary Table S3. Lists of DEGs in *L. rhamnosus* K32 strain treated with cytokines (IL-6, TNFα, IL-8, IL-10) at 1 ng/mL concentration in the medium compared to the GFP. Supplementary Table S4. Orthogroups of genes from representatives of the *Lacticaseibacillus* genus, identified through phylogenetic profiling. Supplementary Table S5. Predicted operons in *L. rhamnosus* strains K32 and R19-3. Supplementary Table S6. Lists of differentially expressed genes (DEGs) in *L. rhamnosus* R19-3 strain treated with cytokines (IL-8, IL-10) at 1 ng/mL concentration in the medium. Supplementary Figure S1. Principal component analysis (PCA) of data from all experiments. Supplementary Figure S2. Global DEGs in *L. rhamnosus* R19-3 resulting from (A) exposure to IL-8, (B) exposure to IL-10 (FC ≥ |2|, FDR < 0.05). Red dots: upregulated genes (UP); blue dots: downregulated genes (DOWN); gray dots: not significant genes (Not Sig). Supplementary Figure S3. Operons of *L. rhamnosus* R19-3 identified using the phylogenetic profiling method, whose gene expression depends on the presence of cytokines (A) IL-8 and (B) IL-10 in the medium.
