# Supplementary material for: Transcriptional Responses of Lacticaseibacillus rhamnosus to TNFα, IL-6, IL-8, and IL-10 Cytokines
Source: Biology (Basel). 2024 Nov 15;13(11):931. doi: 10.3390/biology13110931 (PMC11591797; doi:10.3390/biology13110931)
Supplement: Supplementary file 1 [file biology-13-00931-s001.zip › Supplementary files_v2/Table_S1.docx]

Table S1 - Total reads from RNA-sequencing of *L. rhamnosus* K32 treated with different cytokines.

| **Sample name** | **Concentration, ng/ml** | **Total number of reads** | **Number of reads mapped to the genome** |
| --- | --- | --- | --- |
| Control | 0 | 28 341 299 | 26 825 845 |
| IL-6 | 0.1 | 22 701 133 | 21 703 943 |
|  | 1.0 | 23 615 467 | 22 358 133 |
|  | 10.0 | 32 402 343 | 30 820 937 |
| IL-8 | 0.1 | 16 658 796 | 15 788 944 |
|  | 1.0 | 23 571 644 | 22 444 780 |
|  | 10.0 | 28 394 400 | 27 071 269 |
| IL-10 | 0.1 | 25 982 095 | 24 663 764 |
|  | 1.0 | 20 412 590 | 19 416 474 |
|  | 10.0 | 28 491 486 | 27 055 776 |
| TNFα | 0.1 | 27 363 802 | 26 069 250 |
|  | 1.0 | 15 241 304 | 14 480 642 |
|  | 10.0 | 24 451 092 | 23 254 112 |
