# Supplementary figures and images for: Transcriptional Responses of Lacticaseibacillus rhamnosus to TNFα, IL-6, IL-8, and IL-10 Cytokines
Source: Biology (Basel). 2024 Nov 15;13(11):931. doi: 10.3390/biology13110931 (PMC11591797; doi:10.3390/biology13110931)

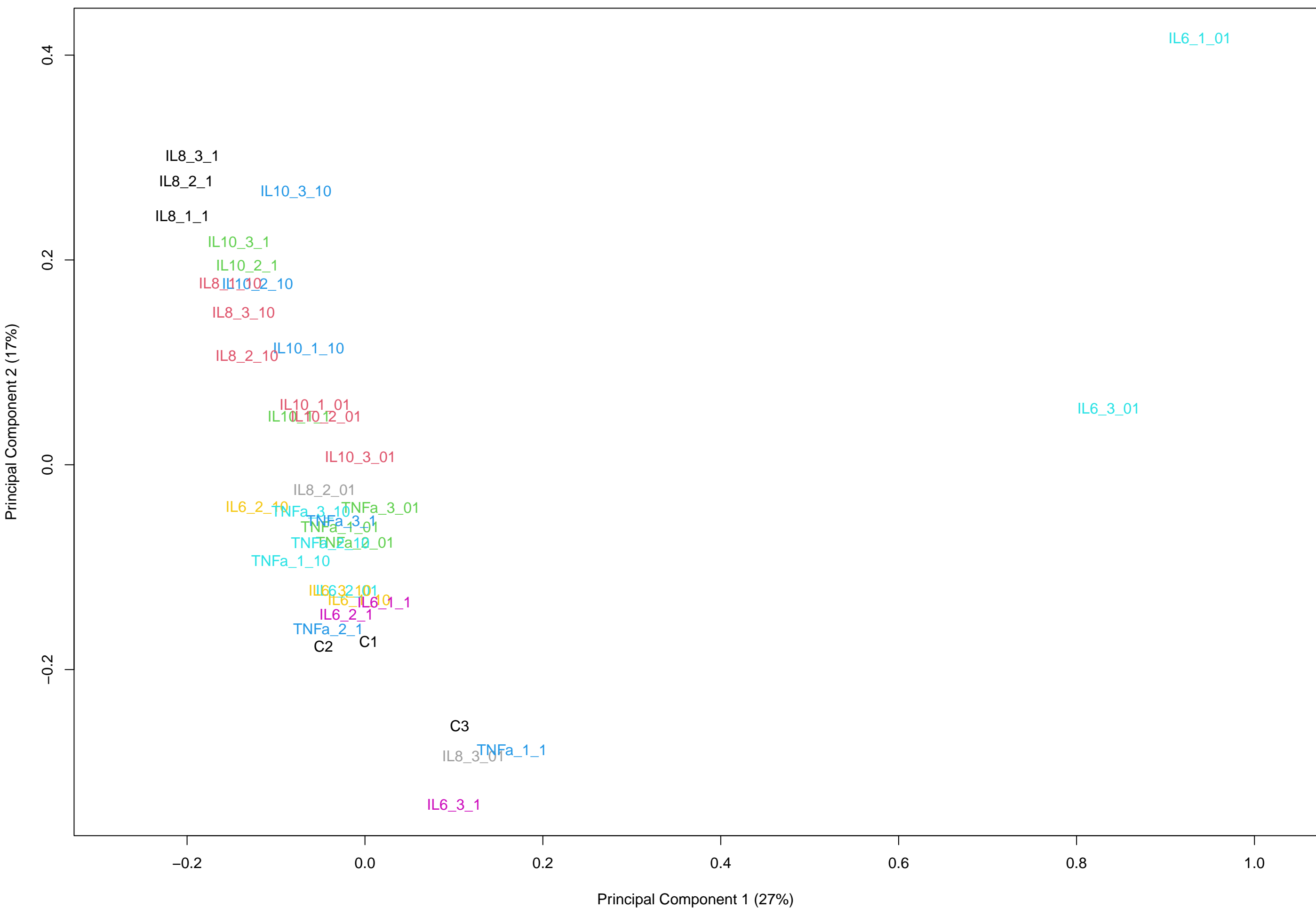

Supplement: Supplementary file 1 [file biology-13-00931-s001.zip › Supplementary files_v2/Figure_S1.pdf]

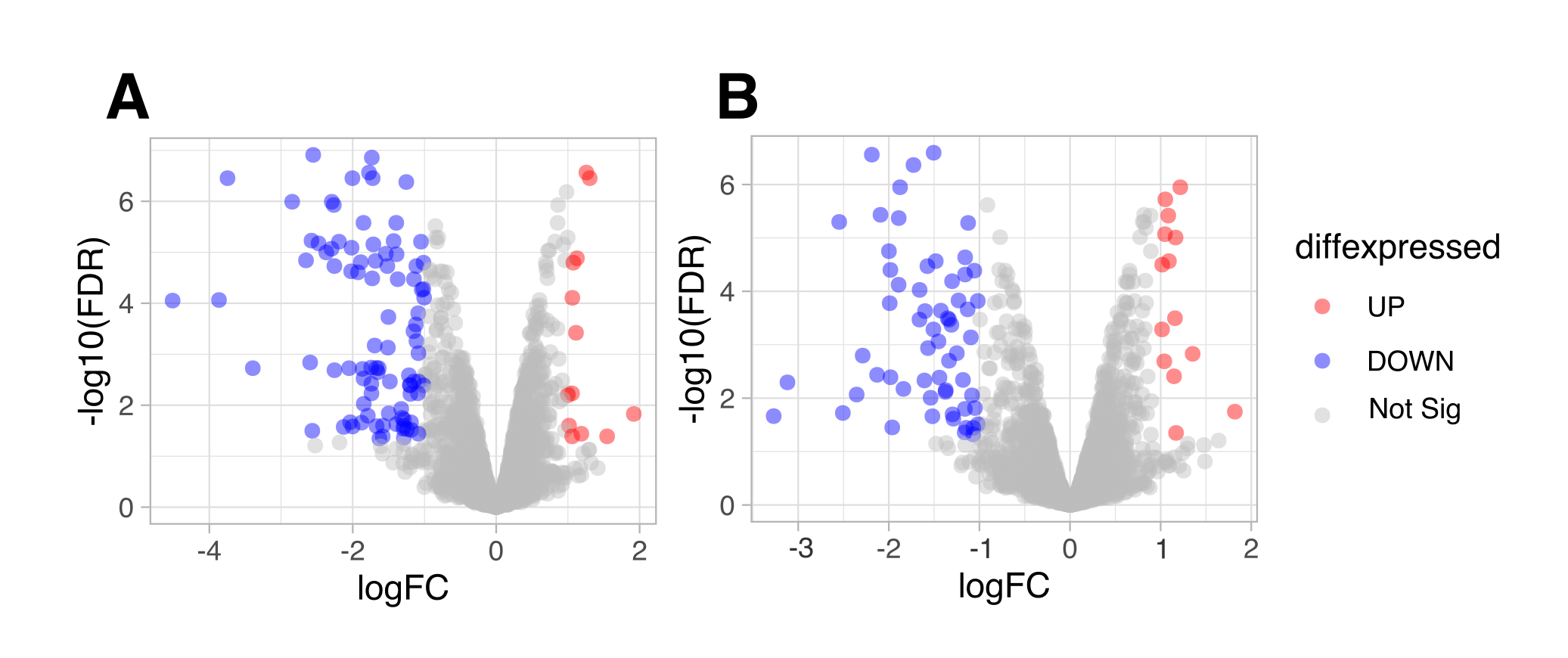

Supplement: Supplementary file 1 [file biology-13-00931-s001.zip › Supplementary files_v2/Figure_S2.png]

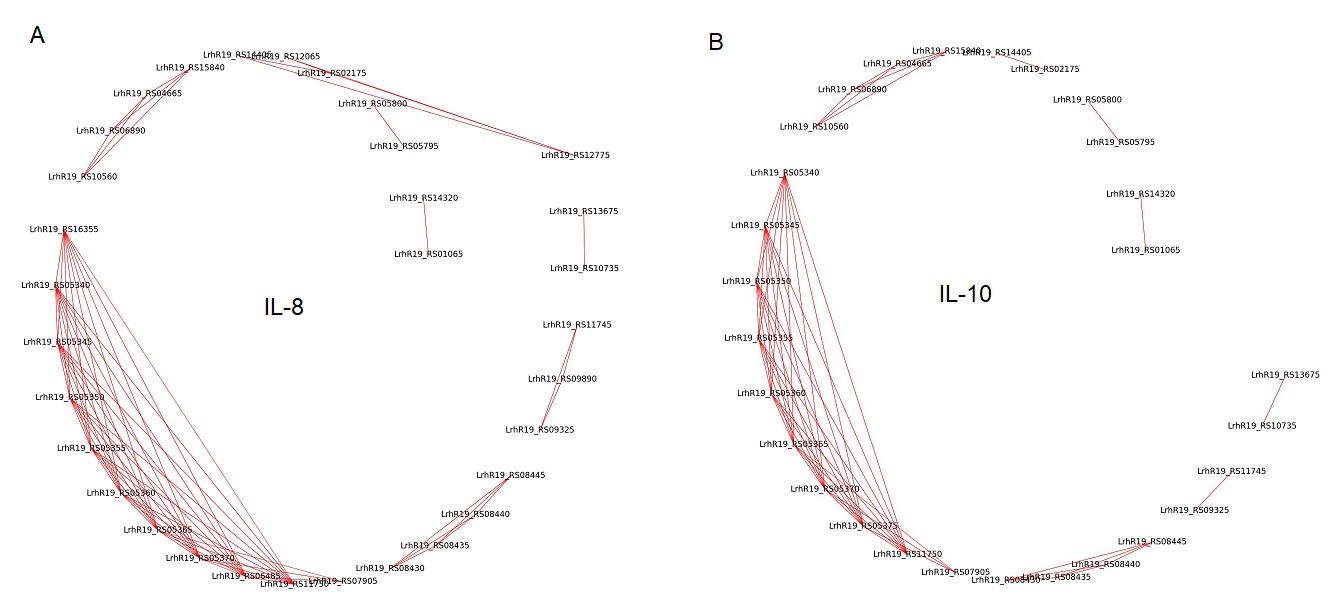

Supplement: Supplementary file 1 [file biology-13-00931-s001.zip › Supplementary files_v2/Figure_S3.jpg]
